# Managing Accident Prevention in Ski Resorts: Participants’ Actual Velocities in Slow Zones

**DOI:** 10.3390/ijerph20075302

**Published:** 2023-03-29

**Authors:** Luis Carus, Xhevrije Mamaqi-Kapllani

**Affiliations:** Faculty of Health and Sport Sciences, University of Zaragoza, 50009 Zaragoza, Spain; mamaqi@unizar.es

**Keywords:** risk management, critical areas, actual maximum velocity, personal characteristics, environmental conditions

## Abstract

Velocity is one of the main factors affecting the kinematic of snow sports’ accidents and the severity of resulting injuries. The aims of the present study were to measure the actual maximum velocities attained by a sample of snow sports participants in slow zones, to compare them to the recommended velocity limits and to assess whether their velocities were in any way related to their personal characteristics and to environmental conditions. Data were drawn from a sample of 1023 recreational skiers and snowboarders during the 2021–2022 winter season at four ski resorts located in the Spanish Pyrenees. Maximum velocity measurements were taken by the authors with a radar speed gun whose precision had been previously validated. Bivariate analysis tests were used to compare the influence that personal characteristics and environmental conditions had on the participants’ maximum velocities. Furthermore, a multivariate analysis was performed. The binary logistic regression was used to distinguish the categories of personal and environmental factors that have the highest probabilities of impact on different segments of velocity. As generally accepted, probability values were two-tailed, and values of 0.05 or less were regarded as statistically significant. Participants’ mean measured maximum velocity (±SD) was 51.61 (±16.14) km/h. A vast majority of the participants in this study traveled in slow zones at actual maximum velocities well over the recommended limits. Multivariate analysis showed that modality and both environmental conditions (visibility and snow quality) were highly significant and can be used to explain the chances of an increase in velocity in slow zones. Further research is needed to investigate causal relationships between skiers and snowboarders’ accidents, injuries and disrespect for velocity limits.

## 1. Introduction

Past research into on-slope ski and snowboard accidents in ski resorts has shown how one of the main factors related to their occurrence, and to the severity of resulting injuries, is the velocity of the participants involved immediately preceding the accidents. These are very often the result of excessive velocity, and the severity of the resulting injuries are likely to increase as velocity increases [1,2,3]. This relationship can be traced back, on the one hand, to the fact that, for a given mass, kinetic energy increases as the square of the velocity. On the other hand, it can be traced back to the fact that high velocity negatively affects the time and distance needed to conduct an adequate response to avoid obstacles or other persons [4,5].

Therefore, as safety is one of the ski resorts’ managers’ main concerns, the control of velocity is a risk-management strategy that, to be effective, requires an understanding of the velocity at which participants are traveling. In particular, this applies in those critical areas of potentially greater risk, the resort-designated “slow zones”, which are often clearly signposted on the slopes and marked on the resorts’ trail maps, indicating that a slow velocity is warranted [6].

Apart from other “slow points”, such as intersections, elevation changes or narrowings that are also usually signposted and marked so as to warrant a slow velocity, these problematic slow zones particularly include areas of collection. In such areas, a number of runs of varying difficulty feed into wide green ones (easiest (≤10° of inclination)) within them to provide access to the lifts or easy access to the bottom of the hill, and also easy areas where most frequently novice participants take their first steps into snow sports [7,8].

These are areas of heavy traffic and potential congestion where skiers and snowboarders of all genders, ages, skill levels and risk-taking behaviors intermix under varying weather, visibility and snow conditions. However, they favor relatively high velocities because the low steepness of these areas seems to encourage users to descend straight in order to maintain a high velocity rather than to perform turns; thus, this might explain that it is on these easy areas that most collisions occur between participants leading to head injuries (the most common type of accident behind lawsuits) [6,9,10,11]. In this sense, Dickson et al. found that no less than nearly 20% of participants in their study recorded their maximum velocity for the whole day in or entering a designated slow zone [6].

All of the above, together with the general inability of skiers and snowboarders, of all skill levels and degrees of experience, to accurately perceive their actual velocity without an external prompt, make slow zones of special interest for preventive measures [6,7,8,11,12,13,14].

In reference to the control of velocity and manner of skiing or snowboarding, the second Rule of the International Ski Federation (FIS), without specifying velocity limits, states that “a skier or snowboarder must move in control. They must adapt their velocity and manner of skiing or snowboarding to their personal ability and to the prevailing conditions of terrain, snow and weather as well as to the density of traffic. In crowded areas or in places where visibility is reduced, skiers and snowboarders must move slowly especially… within areas surrounding ski lifts” (p. 1) [15].

However, for snow sports’ participants, this rule is difficult to comply with because as Dickson et al. put it, “how is a skier or boarder to know what velocity they are actually traveling without an external prompt? This raises the question, how slow is slow?” (p. 3) [6]. This seems to be the reason why in some European resorts posted velocity limits of 30 km/h have been imposed on selected runs, while a number of North American resorts will be implementing a velocity limit of 17 km/h in slow zones [16,17].

In this sense, the past research that has investigated what an appropriate velocity limit would be in slow zones has shown how a motley sample of recreational and expert snow sports’ participants have mainly recommended a velocity limit of 30 km/h, while a sample of professional ski instructors have mainly recommended a velocity limit of 20 km/h, which is a much more conservative recommendation for slow zones than that of the broader public, and which is more in tune with the flat impact test standard for snow sports helmets of 22.3 km/h [6,8,18].

Considering that slow zones may favor high velocity and that past research has abundantly shown how most skiers and snowboarders tend to underestimate their actual velocity [6,7,8,11,12,13,14], we hypothesize that participants in snow sports generally travel in slow zones at an average maximum velocity beyond the recommended velocity limits, which would indicate that the present ski resorts’ strategies to control velocity in these areas are not as effective as would be desirable.

Therefore, given the importance of the subject for the design of accident prevention policies, this exploratory study seeks to extend the insights gained in previous research and to contribute to the existing body of knowledge by, firstly, measuring the actual maximum velocities attained by a sample of participants descending in slow zones. Secondly, by comparing them to the recommended limits. Thirdly, if our hypothesis proved true, by trying to find out whether excesses of velocity are in any way related to personal characteristics (Gender, Modality, Helmet use and Skill level) and to the two environmental conditions (Visibility and Quality of snow) that previous research has shown to have the most significative impact on actual velocity on the slopes [7,11,19,20].

## 2. Materials and Methods

### 2.1. Participants

The data were gathered between December 2021 and March 2022 at four ski resorts located in the Spanish Pyrenees (each of them between 650,000 and 950,000 visitor days per season), from a sample of anonymous recreational male and female skiers and snowboarders of all ages and skill levels, travelling on different slow zones that provided easy access to the lifts or to the bottom of the hill. Pupils taking a ski or snowboard lesson, professionals (instructors, patrollers and maintenance staff) and persons who were in the process of stopping were excluded.

Mean maximum velocity (±SD) of the sample was 51.61 (±16.14) km/h. The maximum measured velocity was 81.7 km/h, for a helmeted, more skilled male skier on a day of good visibility and grippy snow, while the minimum was 14.3 km/h, for an unhelmeted female less skilled skier on a day of poor visibility and wet snow. Mean actual maximum velocity with regard to gender, modality, helmet use, skill level, visibility and quality of snow are displayed in Table 1.

### 2.2. Protocol

Maximum velocity measurements were taken by the authors with a radar speed gun (Stalker ATS II, Plano, TX, USA) with an accuracy of +/−3% of reading, from 1.61 km/h up to more than 300 km/h (the manufacturer’s stated specifications). It could be and was set to automatically compensate for the cosine angle error to display true object velocity (though collinearity was sought as much as possible), and to monitor targets bidirectionally (target moving toward and away the radar). The recorded velocity value (km/h) was the maximum velocity attained during the period the subject was under observation.

We had the radar’s precision previously validated by comparing its results on a slow zone with measurements made in the same zone with a 2019 version of Ski Tracks (Core Coders Ltd., Billingshurst, UK), a GPS-based application for smartphones. The results proved negligible differences in maximum velocity with no systematic bias.

The slow zones under study were fed in all cases by blue runs (easy: between 10° and 22° of inclination). In each of them, observers remained in the same position and velocity measures were taken at estimated distances between 40 and 90 m, well within the clocking distance of 2.8 km stated by the manufacturer. In addition, in order to make the speed measurement process as inconspicuous as possible by avoiding the participants being aware that they were under observation, and so avoiding the measurements being contaminated by the process itself, observers were typically placed behind or below trees, huts, snow guns and lift towers [12].

Two groups of factors were thoroughly examined: the personal characteristics, which comprised gender, modality, helmet use and skill level; and the environmental conditions, which included visibility and snow quality. All of the variables dealt with are categorical and qualitative variables with two or three categories, with the exception of the maximum velocity, which is a continuous one.

The authors were assisted in the observational processes by experienced ski and snowboard Level III-certified instructors who were in charge of recording whether the participants observed were male or female, were a skier or a snowboarder and whether they were wearing a helmet or not. They also graded the observed skiers and snowboarders’ skill levels (low, intermediate or advanced) in compliance with generally accepted criteria [21]. However, in the first stages of statistical analysis regarding skill levels, differences in measured maximum velocity between intermediate and advanced levels turned out to be non-significant and, therefore, the skill levels were then grouped into less skilled (low) and more skilled (intermediate and advanced) [13,22].

The data on environmental conditions were gathered on-site, as velocity measurements were taking place, using the resorts’ scales for visibility (good (+1000 m)/moderate (500–1000 m)/poor (−500 m)) and quality of snow (grippy/icy/wet). They were also checked against those broadcasted in real time through the resorts’ own websites and smartphone applications, with no noticeable differences having been observed.

### 2.3. Statistical Analysis

Given the aforementioned visitor days per season, the sample dealt with was representative, in statistical terms, of an infinite population, with a confidence level of 99.7% and an error of 5%. In addition to a descriptive analysis of the sample, further tests to contrast the main hypotheses regarding the influence of personal characteristics (P. Characs.) and environmental conditions (E. Conds.) on the velocity of participants in the slow zones were chosen in accordance with the scales used to measure the variables considered.

Thus, bivariate analysis tests were used to compare the influence that personal characteristics and environmental conditions had on the participants’ maximum velocities. These included the mean difference test (for variables with two categories) and ANOVA (for variables with more than two categories). Furthermore, a multivariate analysis was performed. The binary logistic regression was used to distinguish the categories of personal and environmental factors that had the highest probabilities of impact on different segments of velocity. In order to perform the logistic regression, the variable “velocity” was converted into a categorical one after quartile analysis, and the categories of personal and environmental variables were grouped into two categories while maintaining the group composition and minimizing the loss of information from the original variable.

Data, on measured velocities as well as on individual characteristics, visibility and snow quality, were processed using SPSS 29.0 for Windows (IBM, Armonk, NY, USA). Probability values were two-tailed, and *p*-values of 0.05 or less were regarded as statistically significant.

## 3. Results

### 3.1. Sample Description, Mean Difference Test and ANOVA

A total of 1023 participants, 61% Male (87% Skiers, 13% Snowboarders) and 39% Female (88% Skiers, 12% Snowboarders), less skilled (34%) and more skilled (66%), and 65% of them wearing helmets, were measured in different environmental conditions of visibility (good 50%, moderate 37%, and poor 13%) and quality of snow (grippy 63%, icy 16%, and wet 21%) in this study.

Personal characteristics are all variables with two categories. Previous to the mean difference test, the normal distribution of velocity for each category had been verified but failure to comply with the normality assumption required the use of non-parametric contrast and so the U-Mann–Whitney Z-test was performed. Results showing that relevant mean differences for velocity are present for gender, modality and skill level are displayed in Table 2.

Environmental conditions are both variables with three categories and so the ANOVA analysis was used to perform multiple comparisons and statistically compare the means. Results of the F statistic of ANOVA showing that there were statistically significant differences for both, visibility and snow quality, are shown in Table 3.

**Figure 1:**
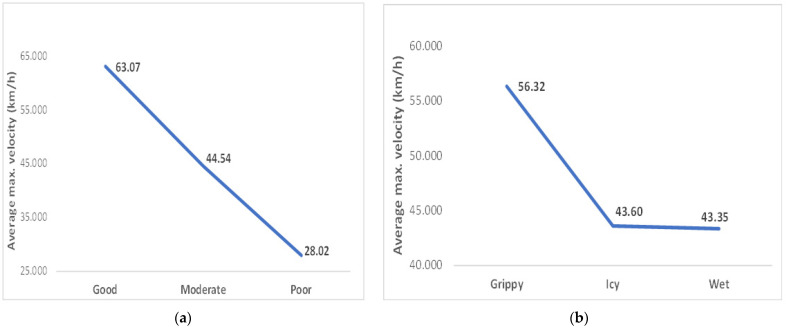
Maximum speed for visibility (**a**) and snow quality (**b**) categories.

As expected, it was observed that the maximum velocity is associated with environmental conditions, in the sense that as the latter improve velocity increases and vice versa. In addition, good visibility encourages participants to increase their velocity more than the quality of the snow itself (Figure 1).

However, although a clear difference between category means could be observed, further analysis was needed to reject the causality of these differences between categories. The results shown in Table 3 do not indicate between which groups this difference occurs and, therefore, an ad hoc test was needed to find out which categories differ. In this sense, the multiple comparison of mean differences between different categories of the variable itself made it possible to know between which categories these differences were significant. For such a purpose, the Scheffe test was performed using the sample size of the harmonic mean (=246.80) for multiple comparisons, since the size of the groups differed. The means of the groups in the homogeneous subsets are displayed in Table 4.

The statistical significance of the mean differences for multiple comparisons was less than the given level, with only one exception between the categories of icy and wet snow quality since the significance was very close to the unity.

### 3.2. Binary Logit Model

A binary logit model was used to assess the variables that affected participants’ velocity under the null hypothesis that personal and environmental variables have no bearing on the maximum velocity in the slow zones and, consequently, on the likelihood of accidents. Therefore, the alternative and the study’s major purpose, which is that personal and environmental factors influence participants’ velocity, will be supported by the null hypothesis being rejected. It means that statistically significant logit coefficient estimations are anticipated in empirical terms equal to zero [23].

In this model, it is assumed that for the observable dichotomous variable, “maximum velocity” (Y), there is a latent variable Y* that represents the likelihood that a participant, subject to a variety of personal characteristics and environmental conditions, will situate below (or equal to) and above the median (Table 5).

The underlying variable Y* in the model estimation process takes the internal categorical values 0 and 1 corresponding to the initial categories 1 and 2 shown in Table 5. The resolution of the logit model is carried out in terms of probability and odds. The probability is represented by the variable Y*:

$${Y^{*}=P}_{i}=1$$

is the probability that the participant travels below or equal to 53.7 km/h, and

$$Y^{*}=1-P_{i}=2$$

is the probability that he or she travels over 53.7 km/h.

The odds are defined as the ratio between the probabilities of the two alternatives:

$$\Omega =\frac{P(Y_{i}=2|x)}{P(Y_{i}=1|x)}=e^{x\beta }$$

where x corresponds to the personal and environmental variables and β are the logit regression coefficients to be estimated.

When the independent variable has no influence, the odds ratio is 1, and the regression coefficient corresponding to it is 0 (not statistically significant). When a rise in the independent variable is accompanied by a rise in the probability of the event, the odds ratio and its corresponding regression coefficient are higher than one, and when a rise in the independent variable causes a fall in the event probability the odds ratio and the corresponding regression coefficient are 1. As a result, the value 1 serves as the standard for interpreting both the coefficients and odds ratio.

The estimation coefficients that show how the independent and dependent variables relate to one another can be interpreted as follows: the change in Y caused by a unitary change in X is represented by the coefficient, which is linked to the independent variable. As a result, it exemplifies how the logarithm’s viability would vary in favor of appropriately classifying participants into each of the two groups of maximum velocities under the influence of the circumstances outlined by personal characteristics and environmental conditions. The model’s constant had little significance. The global fit tests of the model are summarized in Table 6.

The value of the $\chi^{2}$ statistic and its significance indicate a good fit of the model. The 2 Log Likelihood (−2LL) also measures how well the model fits the data; the smaller the value, the better the fit. The Cox and Snell $R^{2}$ is a generalized coefficient of determination used to estimate the proportion of variance of the dependent variable explained by the predictors (independent variables). It is based on the comparison of the Log-Likelihood (LL) for the model to that for a baseline model. Its value, that should oscillate between 0 and 1, in our case was 0.620 which indicated that 62% of the variation in the dependent variable was explained by the variable included in the model.

An important indicator of a good fit of the model to the dataset is the percentage of correctly predicted cases that match the initial clustering (Table 7).

There were only 52 (10.1%) and 54 (10.3%) cases that incorrectly belonged to groups 1 and 2, respectively. Almost 90% of cases were correctly located in each group after estimating the model.

All values regarding variables, estimated coefficients, test indicators, odds ratios and confidence intervals of the estimated model are shown in Table 8. The model’s variables are all categorical, and the categories of the same variable were compared using the last level of each one.

The Wald test affects how the odds should be understood. It is assumed that this coefficient is not zero and that, as a result, the model is useful to depict a particular connection provided that the *p*-value is lower than 0.05, which would disprove the null hypothesis that asserts that it is.

In our case, for category 1, the maximum velocity was affected by various factor categories. Effectively, three of the six variables that were included in the model (modality, visibility and snow quality) presented values for estimated coefficients and odds ratios that could be assessed and statistically analyzed. The other estimates, all based on individual characteristics (gender, helmet use and skill level), had not produced statistically significant results.

## 4. Discussion

Gaining insight into snow sports’ velocity in slow zones is essential to design effective accident prevention policies and to assess means for skiers and snowboarders’ information and protection. Measured actual velocities, in combination with data on snow sports participants’ personal characteristics and environmental conditions, could provide those responsible for managing risk in ski resorts with valuable information on how to design accident prevention strategies for slow zones.

The objective of the present study was to enhance the knowledge of snow sports participants’ velocity through trying to understand skiing and snowboarding velocity in slow zones by investigating the maximum velocity at which recreational skiers and snowboarders travel on the zones and how individual personal characteristics and environmental conditions affect the velocity, with the intention that the results obtained will help stakeholders (participants, resort managers, educators, etc.) to make informed decisions on how to manage behavior on slow zones for the safety of all.

This study, carried out at four ski resorts located in the Spanish Pyrenees, found that typical velocities in slow zones were consistent across all four resorts. They are what would be referred to as mid-sized resorts in that they have between 650,000 and 950,000 visits annually. This suggests that similar slopes would generate similar velocities regardless of location.

The main result is that 99.5% and 89.2% of the sample traveled in slow zones at velocities over the recommended limits of 20 km/h and 30 km/h, respectively. Participants traveled at an average maximum velocity of 51.61 km/h which more than doubled the first, more conservative, recommended velocity limit and is still far beyond the more venturesome second one. It is slightly higher than those previously registered by Dickson et al. in slow zones (47.3 km/h) [6], and by Bailly et al. on green slopes (48.4 km/h) [11], differences that may well be due to varying proportions in personal characteristics of the respective samples, and/or to conspicuously different environmental conditions when the respective measurements were made.

With respect to personal characteristics, the mean maximum velocity in slow zones significantly differed between the respective categories of gender, modality and, above all, skill level. In this sense, the velocity of males was higher than the velocity of females, by a mean of 12.6 km/h; skiers were, by a mean of 9.1 km/h, faster than snowboarders; and the velocity of more skilled participants was higher than that of less skilled participants, by a noticeable mean of 28.5 km/h.

In reference to the environmental conditions, the ANOVA test showed how the mean maximum velocity significantly differed between the categories that make up the variable visibility. Indeed, when compared good with moderate and poor visibility, the mean maximum velocity decreased by 18.5 and 35 km/h, respectively. However, this was not entirely the case for the quality of snow because, although the statistical significance of the mean differences for comparisons to grippy snow was less than the given significance level, between the categories of icy and wet snow, the latter was very close to the unity.

The tests carried out for the estimated logit model showed a very good fit of the dataset. Under the influence of personal and environmental factors, 89.6% of the cases among adjustment indicators were accurately predicted according to estimations. According to the model, it turned out that three out of the six variables that had been included were significant in describing the level of velocity above the median. As a result, its significance can be generalized to all participants in the target population (snow sports visitors travelling in slow zones), regardless of how the mean differences affected each individual in the bivariate analysis for these parameters.

Effectively, modality and both environmental conditions (visibility and snow quality) were highly significant and could be used to explain the chances of an increase in velocity in slow zones. According to the predicted coefficients for these three variables, which were all positive, the odds ratio for every participant in category 1 (>53.7 km/h) was greater than the unity (exponential of β). Therefore, the positive coefficients showed that an increase in skiers over snowboarders within the slow zones, as well as an improvement in environmental conditions, increased the likelihood of the participants travelling at a velocity above the median and, consequently, the average (51.61 km/h).

It is noteworthy that environmental factors showed the greatest influence. The likelihood of travelling above the median in good and moderate visibility is 7.32 and 5.94 times, respectively, greater than travelling above the median in poor visibility. A similar pattern to that of visibility was also shown in the case of snow quality, although with a more significant impact. In this case, the probability of sliding above the median on grippy and icy snow was 8.81 and 3.04 times, respectively, greater than sliding on wet snow.

Both the mean maximum actual velocity of the whole sample and that of the different groups of participants would indicate that in fact the present ski resorts’ strategies to control velocity in these areas, such as posting slow velocity recommendations and velocity limits or ski patrol surveillance, be it either because signage is deliberately disregarded [24], because of a lack of knowledge of the recommendations on velocity control [25], or because snow sports enthusiasts are simply unable to accurately assess at what velocity they are actually travelling without exogenous information [6], are not as effective as would be desirable and that new more effective strategies to control velocity in slow zones should be implemented.

In this sense, education can be a particularly suitable policy for modifying behaviors in slow zones in as much as it can help snow sports participants to become more conscious of their own demeanor, of its possible repercussions and of how to improve it [6]. Since the behavioral patterns of skiers and snowboarders are closely connected to their knowledge of existing safety rules, in order to reduce the quantity of accidents in slow zones and their severity it would be instrumental that they were educated in the importance of adapting their velocity both to their personal characteristics and to the existing environmental conditions [25,26].

Therefore, all the stakeholders (resorts, snow sports clubs and schools, parents, instructors, practitioners, etc.) should be involved in comprehensive snow-sports education and take action to create awareness and understanding of how undue velocity increases the likelihood of undergoing crashes in slow zones, and of the benefits of being able to accurately assess at what velocity they actually travel. For such purpose, Ruedl et al. advocate for training to take place in velocity self-awareness, which “could easily be integrated into ski education courses” (p. 122) [13].

Although the work by Pinelly et al. on velocity control in ski resorts, in which a procedure was implemented to ingeniously influence behaviors by way of rousing mental representations, provided encouraging conclusions [27], given that skiers and snowboarders experience real difficulty in gauging their actual velocity without external prompts [6,7,8,11,12,13,14], the prevention of velocity-related accidents in slow zones should include the avoidance of excessive velocity through further strategies.

In this respect, posted electronic velocity limits combined with radar boards in slow zones could not only act as external prompts for skiers and snowboarders to know certainly the exact velocity at which they are travelling with reference to the given velocity limit but they would also allow for the adjustment in velocity limits according to the degree of congestion and the prevailing environmental conditions. Furthermore, new gadgets based on GPS technology (e.g., smartphones, navigators or smart googles) are supplementary devices that can both punctually inform skiers and snowboarders about their actual velocity and, more importantly, help them to enhance their skill to assess velocity and their awareness of their own behavior [8,14]. Therefore, those responsible for snow sports participants’ apprenticeship should include the operation and functionality of such devices in their education programs.

Finally, the present research results are somewhat constrained in as much as the data were gathered from the observation of recreational snow sports participants alone and so excluded professionals what means that, had the latter actual maximum velocities been included, presumably the average maximum velocity in slow zones would have been even higher [8]. However, our results on measured maximum velocities appear to be in line with those obtained both in Europe and North America [6,11].

Our results are also limited because velocity can be affected by other factors additional to those that anonymity allowed for this study (age, weight, risk-taking behavior, etc.). In this sense, since independent variables increase their influence by interacting with one another, further research is needed to develop a multi-year database which included all the critical variables that might have a significant impact on velocity and, therefore, on potential accidents.

This study is also potentially limited by the fact that data were obtained from snow sports participants at resorts located in the same mountain range in one country and may not apply to others.

Although the results may be considered useful to generate new hypotheses on snow sports participants’ maximum velocities in slow zones, further research is needed to investigate the causal relationships between skiers and snowboarders’ injuries and disrespect for velocity limits.

However, this study would provide concerned parties with profitable data so as to gain insight into some of the significant aspects of the issues to be fixed through a variety of suitable educational and preventive strategies.

## 5. Conclusions

We found that an overwhelming majority of the participants in this study traveled in slow zones at actual maximum velocities well over the recommended limits. We also found that personal characteristics and environmental conditions seem to affect actual maximum velocities of skiers and snowboarders while travelling within slow zones boundaries. Modality and, particularly, visibility and the quality of snow were significantly associated with velocity differences. These findings suggest that further and more effective strategies for the communication of velocity limits in slow zones, having male skiers as their main target, must be implemented. A reinforced understanding of velocity in slow zones is helpful to understand accidents and their ensuing damages, to assess protective devices and to design effective prevention strategies. Besides, having snow sports participants informed about the benefits of observing velocity limits in slow zones is a key matter for prevention purposes.

## Figures and Tables

**Table 1:** Personal characteristics, environmental conditions and mean values (±SD) of measured maximum velocities.

P. Characs./E. Conds.	N (%)	Mean Measured Maximum Velocity (km/h)
GENDER		
Male	618 (61)	56.62 ± 15.98
Female	405 (39)	43.98 ± 13.11
MODALITY		
Ski	898 (87)	52.72 ± 16.24
Snowboard	125 (13)	43.68 ± 12.91
HELMET USE		
Yes	660 (65)	51.98 ± 16.06
No	363 (35)	50.95 ± 16.26
SKILL LEVEL		
Less skilled	346 (34)	32.73 ± 5.12
More skilled	677 (66)	61.26 ± 10.24
VISIBILITY		
Good	508 (50)	63.07 ± 10.71
Moderate	383 (37)	44.54 ± 11.16
Poor	132 (13)	28.03 ± 3.18
QUALITY OF SNOW		
Grippy	648 (63)	56.37 ± 16.13
Icy	166 (16)	43.61 ± 15.28
Wet	209 (21)	43.35 ± 9.73
TOTAL	1203 (100)	51.61 ± 16.14

**Table 2:** Contrast of mean differences of maximum velocity according to personal characteristics.

Dependent Variable: Maximum Velocity	K-S * Normal Distribution Test (*p*-Value) *	Mean Difference	Z-Test of U-Mann Whitney *
P. Factors			
Gender			
1-Male	0.183 (0.00)	12.64	−12.83 **
2-Female	0.163 (0.00)		
Modality 1-Ski 2-Snowboard	0.198 (0.00) 0.125 (0.00)	9.04	6.65 **
Helmet use			
1-Yes	0.065 (0.00)	1.03	1.15
2-No	0.139 (0.00)		
Skill level 1-Less skilled 2-More skilled	0.131 (0.00) 0.085 (0.00)	−28.53	−25.78 **

* Under the null hypothesis the differences between categories is zero. The normal distribution contrast in each category with *p*-value = 0.00 rejects the null hypothesis of normal distribution. ** Statistical significance < 0.001.

**Table 3:** ANOVA multiple comparison among categories of environmental conditions.

	1	2	3	F-Anova *p*-Value
**Visibility**	Means for maximum velocity subset (α = 0.05)			
1-Good (N = 332)	63.07			<0.001 *
2-Moderate (N = 383)		44.54		<0.001 *
3-Poor (N = 508)	28.03			<0.001 *
**Quality of snow**	Means for maximum velocity subset (α = 0.05)			
1-Grippy (N = 648)	56.32			<0.001 *
2-Icy (N = 166)	43.60			<0.001 *
3-Wet (N = 209)	43.35			<0.001 *

* Statistical significance of mean differences among groups (F-anova *p*-value < 0.001).

**Table 4:** Means differences of maximum velocity according to environmental conditions categories.

Dependent Variable: Maximum Velocity Environmental Conditions		Means Differences (I–J)	SE	Statistical Significance
**Visibility (I)**	**Visibility (J)**			
1-Good	2-Moderate	18.53	0.69	<0.001 *
	3-Poor	35.04	1.00	<0.001 *
2-Moderate	1-Good	−18.53	0.69	<0.001 *
	3-Poor	16.51	1.03	<0.001 *
3-Poor	1-Good	−35.04	1.00	<0.001 *
	2-Moderate	−16.51	1.03	<0.001 *
**Quality of snow (I)**	**Quality of Snow (J)**			
1-Grippy	2-Icy	12.72	1.29	<0.001 *
	3-Wet	12.97	1.18	<0.001 *
2-Icy	1-Grippy	−12.72	1.29	<0.001 *
	3-Wet	0.24	1.88	0.986
3-Wet	1-Grippy	−12.97	1.18	<0.001 *
	2-Icy	−0.24	1.55	0.986

* Statistical significance (*p*-value < 0.001).

**Table 5:** Codification of maximum velocity as a categorical variable.

Maximum Velocity (Y)	Cases	%
Category 1 Maximum velocity ≤ 53.7 km/h	516	50.4
Category 2 Maximum velocity > 53.7 km/h	507	496
Total	1023	100

**Table 6:** Estimated model fit.

Indicators of Global Fit	Values	*p*-Value
$\chi^{2}$	977.23	0.000 **
−2LL	140.86	
Cox Snell $R^{2}$	0.620	

** Statistical significance < 0.001.

**Table 7:** Predicted case table.

Maximum Velocity Categories			Percentage of Correct Prediction
Observed Cases	Predicted Cases *		
	**1**	**2**	
0 ≤ 53.7	464	52	89.9
1 > 53.7	54	453	89.3
Global %			89.6%

* The cut value is 0.5. The predicted probability is for category 1.

**Table 8:** Estimated coefficients, significance, odds ratio and confidence intervals (CI).

P. Factors/E. Factors	β	SE	Wald’s Test	Exp β	CI (95%)
Gender (1 = male)	−1.046	0.315	0.967	0.351	0.026–0.088
Modality (1 = ski)	1.703	0.369	1.696 **	5.490	2.664–11.311
Helmet use (1 = Yes)	−0.151	0.254	0.608	0.850	0.523–1.132
Skill level (1 = less skilled)	−1.063	0.371	0.483	0.345	0.000–0.090
Visibility (1 = good)	1.991	0.378	10.49 **	7.321	7.017–10.365
Visibility (2 = moderate)	1.614	0.356	7.981 **	5.942	5.100–9.865
Quality of snow (1 = grippy)	2.177	0.283	7.895 **	8.819	5.734–9.876
Quality of snow (2 = icy)	1.251	0.397	1.985 **	3.049	1.143–3.765
Constant	−9.932	126.5	8.349 **	----	------

** Statistical significance < 0.001.

## Data Availability

The data presented in this study are available in Dataset S1: Records (XLSX).

## Supplementary Materials

The following supporting information can be downloaded at: https://www.mdpi.com/article/10.3390/ijerph20075302/s1, Dataset S1: Records (XLSX).
